# Detection and Quantification of Klebsiella pneumoniae in Fecal Samples Using Digital Droplet PCR in Comparison with Real-Time PCR

**DOI:** 10.1128/spectrum.04249-22

**Published:** 2023-06-12

**Authors:** Junxia Feng, Xiaohu Cui, Bing Du, Hanqing Zhao, Yanling Feng, Jinghua Cui, Chao Yan, Lin Gan, Zheng Fan, Tongtong Fu, Ziying Xu, Zihui Yu, Rui Zhang, Shuheng Du, Ziyan Tian, Qun Zhang, Guanhua Xue, Jing Yuan

**Affiliations:** a Department of Bacteriology, Capital Institute of Pediatrics, Beijing, China; b School of Biological Sciences, University of Edinburgh, Edinburgh, United Kingdom; University of Cincinnati

**Keywords:** droplet digital PCR, real-time PCR, *Klebsiella pneumoniae*, quantification

## Abstract

This study aimed to develop a rapid and sensitive droplet digital PCR (ddPCR) assay for the specific detection of Klebsiella pneumoniae in fecal samples, and to evaluate its application in the clinic by comparison with real-time PCR assay and conventional microbial culture. Specific primers and a probe targeting the K. pneumoniae hemolysin (*khe*) gene were designed. Thirteen other pathogens were used to evaluate the specificity of the primers and probe. A recombinant plasmid containing the *khe* gene was constructed and used to assess the sensitivity, repeatability, and reproducibility of the ddPCR. Clinical fecal samples (*n *= 103) were collected and tested by the ddPCR, real-time PCR, and conventional microbial culture methods. The detection limit of ddPCR for K. pneumoniae was 1.1 copies/μL, about a 10-fold increase in sensitivity compared with real-time PCR. The ddPCR was negative for the 13 pathogens other than K. pneumoniae, confirming its high specificity. Clinical fecal samples gave a higher rate of positivity in the K. pneumoniae ddPCR assay than in analysis by real-time PCR or conventional culture. ddPCR also showed less inhibition by the inhibitor in fecal sample than real-time PCR. Thus, we established a sensitive and effective ddPCR-based assay method for K. pneumoniae. It could be a useful tool for K. pneumoniae detection in feces and may serve as a reliable method to identify causal pathogens and help guide treatment decisions.

**IMPORTANCE** Klebsiella pneumoniae can cause a range of illnesses and has a high colonization rate in the human gut, making it crucial to develop an efficient method for detecting K. pneumoniae in fecal samples.

## INTRODUCTION

Klebsiella pneumoniae, a capsulated Gram-negative bacterium, can cause urinary tract infections, pneumonia, bloodstream infections and septicemia in humans (1, 2). K. pneumoniae has lately gained prominence as a major opportunistic pathogen, second only to Escherichia coli. K. pneumoniae can gain genetic traits to become hypervirulent (3). Furthermore, because of its high yield of extended-spectrum β-lactamase and carbapenems, K. pneumoniae exhibit high levels of antibiotic resistance. It was designated a “critical threat” bacterium by the World Health Organization in 2017 (4, 5). K. pneumoniae is found in the outdoor environment, including in soil and surface waters (6, 7), but it is also widely distributed on human mucosal surfaces, including of the nasopharynx, respiratory tract, and gastrointestinal tract. Research has showed that for hospitalized patients, K. pneumoniae colonization rates in the colon were as high as 23% in the intensive care unit of a hospital in the United States (8). In a nonhospital setting, substantial gut colonization of K. pneumoniae from 18.8% to 87.7% has also been found in some Asian countries, including Malaysia, Singapore, China, Thailand, etc. (9). Thus, a rapid, accurate, and sensitive method for the detection of K. pneumoniae in fecal samples would be helpful to the monitor of K. pneumoniae abundance, and the control of K. pneumoniae dissemination.

The traditional bacterial culture method was previously widely employed as the gold standard for detecting K. pneumoniae, but it is time-consuming and has low specificity. Compared with microbial culture, PCR showed superior sensitivity, specificity, and timeliness. Real-time PCR is a rapid and commercialized molecular diagnostic tool applied in the detection of a wide range of microorganisms (10–13), including K. pneumoniae, but the accuracy of real-time PCR results relies to a great extent on a standard curve of known concentrations, while low sensitivity and reproducibility limit its clinical application.

Droplet digital PCR (ddPCR) is a potential alternative to conventional real-time PCR for microorganism detection. It is a new DNA quantitation technology that detects the absolute copy numbers of a target without requiring a calibration curve. When performing reactions, the ddPCR mixture is separated into thousands of small-volume reaction droplets, then endpoint PCR is conducted in each droplet individually. After the run, the number of positive and negative reactions is counted. Using Poisson binomial distributions, the absolute copy number of a template can be calculated because the number of templates is positively correlated with the number of positive droplets. ddPCR has been widely applied in many laboratories and for diagnosis of various clinical infectious diseases because of its high sensitivity and accuracy (14–16).

**FIG 1:**
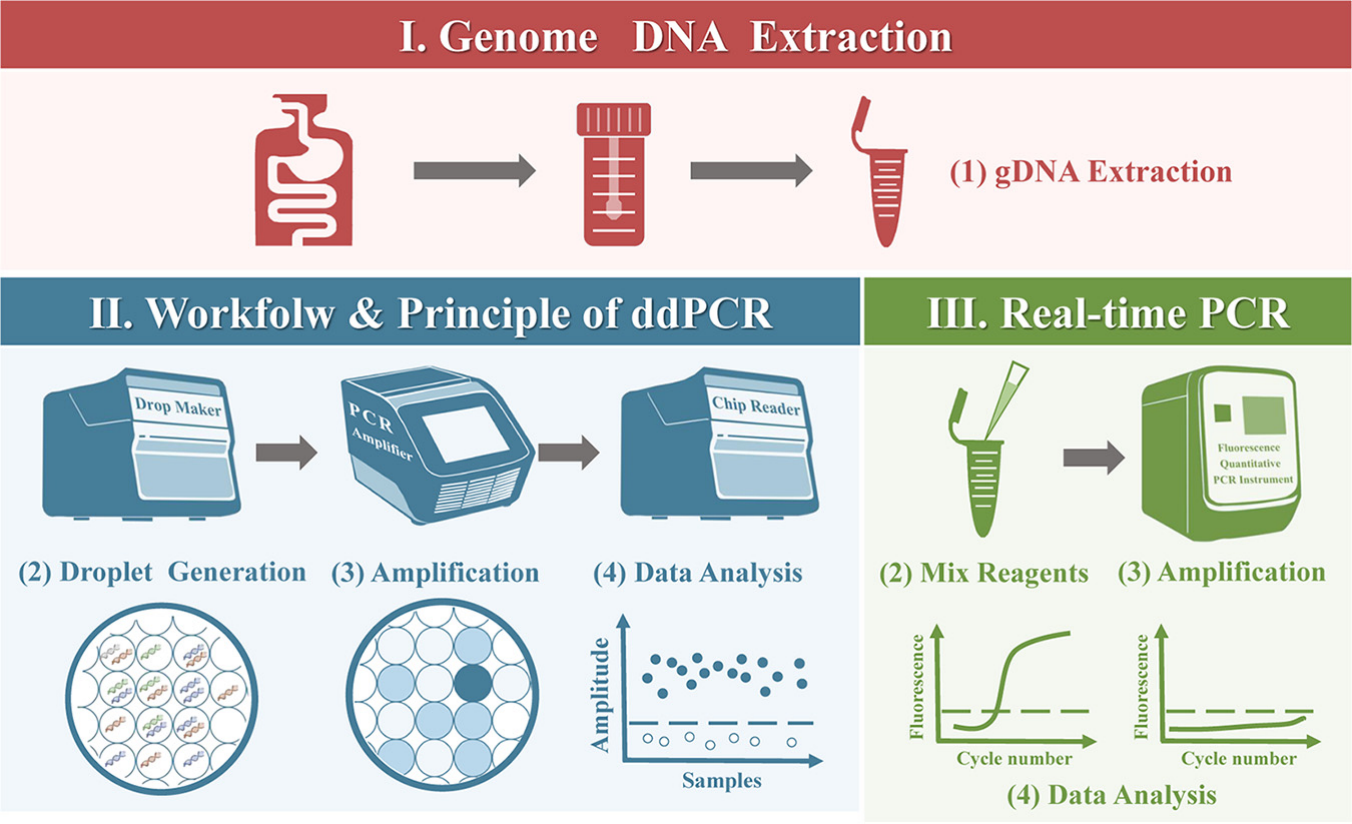
Schematic diagram of detection workflow for K. pneumoniae using ddPCR and real-time PCR assays. The workflow of ddPCR consists of four stages: gDNA extraction, droplet generation, amplification, and data analysis. The workflow of real-time PCR consists of four parts: gDNA extraction, mix reagents, amplification, and data analysis. gDNA: genomic DNA.

In the present study, a sensitive and effective ddPCR method for detection of K. pneumoniae in feces was constructed. To evaluate the application of this method, clinical fecal samples were collected and used for a comparison between ddPCR and real-time PCR methods. The workflow is shown in Fig. 1.

## RESULTS

### ddPCR assay development.

**FIG 2:**
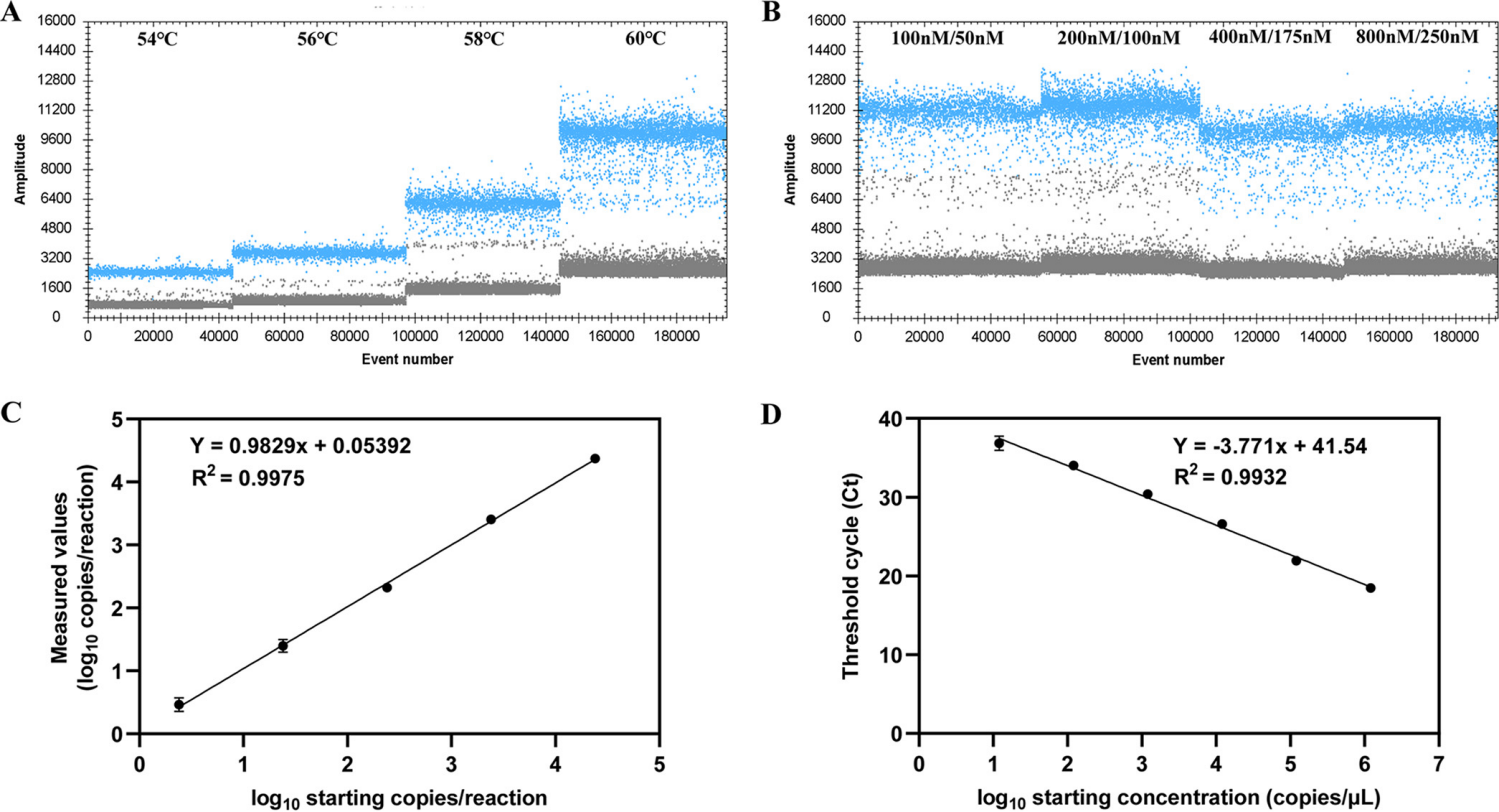
(A) Temperature gradient for ddPCR. (B) Primer/probe concentration at 60°C. (C) Limit of detection for ddPCR. (D) Limit of detection for real-time PCR. Positive and negative droplets are denoted in blue and black, respectively.

To determine the optimum ddPCR assay reaction conditions for the quantitative detection of K. pneumoniae, four elongation temperatures (from 54 to 60°C) were tested, as well as different primer/probe concentrations. Considering the droplets positioned between the positive and negative populations, and the positively and negatively separated droplets. The elongation temperature was set to 60°C, and the primer/probe concentration was 800 nM/250 nM (Fig. 2) in the subsequent ddPCR assay of K. pneumoniae.

Recombinant plasmid pUC57-*khe* was 10-fold diluted to different concentrations (1.2 × 10^4^ to 1.2 copies/μL) to serve as the templates for the linear regression curve construction of the ddPCR assay. The limit of detection and limit of blank was also determined. As shown in Fig. 2, we observed strong linear correlation between staring copies and measured value in ddPCR assay of K. pneumoniae (R^2^ = 0.9975, y = 0.9829× + 0.05392). The limit of detection by ddPCR was 1.1 copies/μL, while the limit of blank by ddPCR was 0.8 copies/μL.

### Real-time PCR assay development.

Ten-fold dilutions of recombinant plasmid pUC57-*khe* (1.2 × 10^6^ to 1.2 × 10^1^ copies/μL) were detected to construct the calibration curve of real-time PCR. The standard curve was plotted by the cycle threshold (Ct) values of the real-time PCR assays against the corresponding logarithm (base 10) of the concentrations of plasmid dilutions. According to the result, real-time PCR showed good linearity with each dilution for plasmid pUC57-*khe* (R^2^ = 0.9932, y = −3.771× + 41.54). The limit of detection by real-time PCR is 10 copies/μL, while the limit of blank by real-time PCR was 7.5 copies/μL.

### Specificity comparison of ddPCR and real-time PCR.

**FIG 3:**
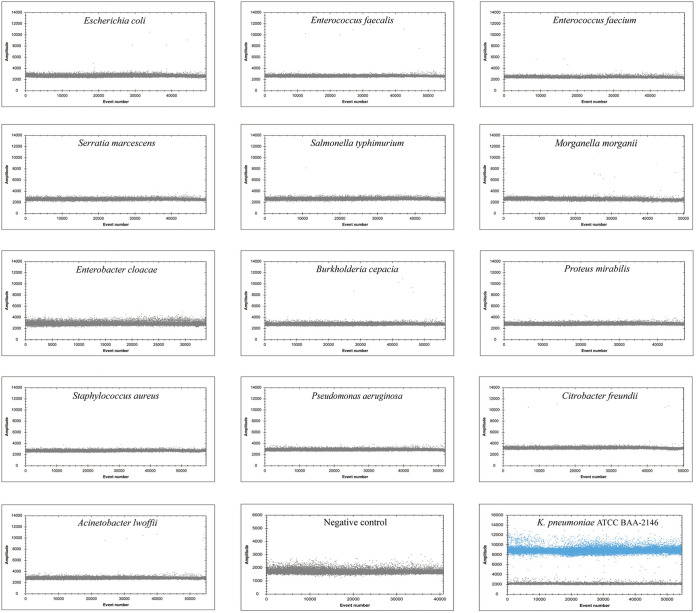
Specificity assay of ddPCR. The DNA of 14 strains was used to perform ddPCR. DNase-free water was used as the negative control. K. pneumoniae ATCC BAA-2146 was used as the positive-control strain. Droplets that were positive and negative are indicated in blue and black, respectively.

The genomic DNA of 14 bacterial strains was extracted and detected by ddPCR and real-time PCR assays for K. pneumoniae. The positive-control strain K. pneumoniae ATCC BAA-2146 was detected by ddPCR, while no positive droplet was observed for the other 13 strains or for the negative control (Fig. 3), suggesting that the ddPCR assay was specific for K. pneumoniae. Consistently, in real-time PCR assay, a positive Ct value (29.2) was detected only for K. pneumoniae ATCC BAA-2146 among the 14 test samples (see Table S1 in the supplemental material).

### Repeatability and reproducibility of the ddPCR assay.

To evaluate the repeatability and reproducibility of the ddPCR assay, various concentrations of pUC57-*khe* recombinant plasmid were tested in triplicate for ddPCR reactions. The intraassay coefficients of variation (CV) of different gradients ranged from 0.061 to 0.216, and the interassay CV ranged between 0.091 and 0.255 (Table 1), suggesting that the ddPCR assay had excellent repeatability and reproducibility.

**TABLE 1:** Repeatability and reproducibility assays of ddPCR in detection of K. pneumoniae^*a*^

Intraassay variation			Interassay variation		
Mean concn (copies/reaction)	SD	CV	Mean concn (copies/reaction)	SD	CV
2595.4	157.8	0.061	2,382.9	369.5	0.155
214.1	21	0.098	207.0	18.9	0.091
23.9	2.8	0.116	27.9	6.2	0.224
2.9	0.6	0.216	3.3	0.4	0.125
1.1	0.1	0.110	1.3	0.3	0.255

a SD, standard deviation; CV, coefficient of variation.

### Performance of ddPCR versus real-time PCR assays with clinical samples.

**FIG 4:**
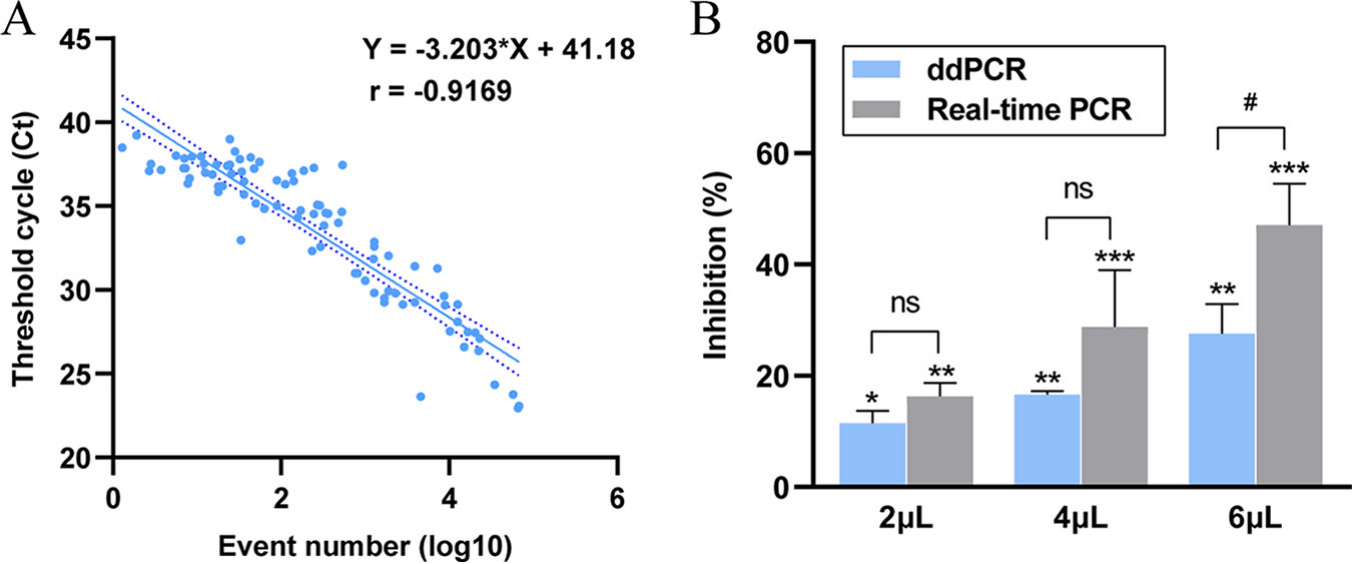
(A) Correlation between real-time PCR and ddPCR. Event numbers for ddPCR are plotted on the *x* axis and the threshold cycle for real-time PCR on the *y* axis. (B) Effect of fecal extract on quantitative detection of K. pneumoniae by real-time PCR and ddPCR. *, *P ≤ *0.05; **, *P ≤ *0.01; and ***, *P ≤ *0.001 after comparison with the inhibition control (DNase-free water). #, *P ≤ *0.05 after comparison ddPCR with real-time PCR. ns, not significant.

To assess the potential clinical application of our ddPCR assay, first, sample size was calculated according to the preliminary experimental results using PASS software; the parameters value of power, alpha, and area under the curve (AUC) were set, the required sample number was calculated to be 102. Then, 103 stool samples were collected. K. pneumoniae was detected in these samples by ddPCR, and the results were compared with those obtained by real-time PCR and microbial culture. Of the 103 stool samples, 99 were found to be positive for K. pneumoniae by ddPCR assay, 91 were positive by real-time PCR assay, and 83 were positive by culture. The clinical sensitivity of ddPCR assay was calculated to be 100% while the clinical sensitivity of real-time PCR assay was 98.8% (Table 2). There was no significant difference between ddPCR and real-time PCR assay (*P* > 0.05). Samples with positive results for both ddPCR and real-time PCR were used for linear regression and correlation analysis, which showed that the log event number of ddPCR decreased with the increase of the Ct value from real-time PCR (the Pearson correlation coefficient was −0.9169) (Fig. 4A).

**TABLE 2:** Comparison of performance between ddPCR, real-time PCR, and fecal culture in detection of K. pneumoniae in clinical fecal samples

Detection method	ddPCR positive	ddPCR negative	Real-time PCR positive	Real-time PCR negative
Culture positive	83	0	82	1
Culture negative	16	4	9	11
	ddPCR clinical sensitivity: 100%		Real-time PCR clinical sensitivity: 98.8%	

### Inhibition of residual matrix on ddPCR and real-time PCR assays.

To identify whether the residual matrix of fecal sample affect the detection efficiency of ddPCR and real-time PCR, we mixed the same quantity of DNA from K. pneumoniae strain ATCC BAA-2146 with different amounts of extract of fecal sample to prepare spiked samples. The outcomes demonstrated that fecal extract hindered the quantitative detection of K. pneumoniae for both ddPCR and real-time PCR assays. The inhibition effect increased with the amount of fecal extract applied. Compared to real-time PCR, the inhibition rate of fecal extract (6 μL) using ddPCR was significantly lower for detection of K. pneumoniae (Fig. 4B).

## DISCUSSION

K. pneumoniae can cause various diseases and easily develops resistance to most antibacterial drugs. Therefore, curing K. pneumoniae-caused infections is becoming increasingly difficult. Early detection is thus essential for timely clinical diagnosis and effective treatment. The conventional detection method, bacterial culture, is slow and has low detection sensitivity for K. pneumoniae. Development of molecular diagnostic techniques has substantially enhanced the efficiency of pathogen detection. Real-time PCR is used as a daily diagnostic tool to check for pathogens (17) due to its good sensitivity and specificity. However, there are some limitations to its use; when the concentration of the sample being tested is very low, real-time PCR may show poor accuracy. Additionally, a standard curve is required for the quantification of K. pneumoniae in each real-time experiment, resulting in a more complex and time-consuming experimental process. Furthermore, the selection of reference standards plays a decisive role in real-time PCR results; an inappropriate reference standard may affect their accuracy, and hence the accuracy of diagnosis.

ddPCR, which is a method that quantifies the absolute copy number of a target DNA, is more sensitive and repeatable than real-time PCR (18, 19) for the detection of the pathogen K. pneumoniae in fecal samples; ddPCR may provide a more simple and sensitive technique. ddPCR has been widely applied to the quantification of various pathogens, such as Mycobacterium *turberculosis* (20, 21), *Ureaplasma* spp. (22), Verticillium dahliae, Verticillium *longisporum* (23), hepatitis B virus (24), and severe acute respiratory syndrome coronavirus 2 (SARS-CoV-2) (25). In recent work, ddPCR showed better detection accuracy and sensitivity for the quantification of SARS-CoV-2 nucleic acid in clinical samples and the diagnosis of patients with COVID-19 than real-time PCR (26, 27). Compared with real-time PCR, ddPCR also has certain limitations, such as smaller sample processing volume than real-time PCR, higher false-positive rate of ddPCR (28), and higher cost due to the requirement of more expensive equipment and reagents than real-time PCR. Meanwhile, the cost of ddPCR is higher than traditional bacterial culture, but much lower than that of metagenomic next-generation sequencing.

In the present study, a ddPCR method was established for the detection of K. pneumoniae in fecal samples. The hemolysin gene of K. pneumoniae, which is often used for the detection of K. pneumoniae in various clinical samples by different methods (29, 30), was chosen as the target gene. Then, we designed specific primers and a probe, screened optimum elongation temperature and primer/probe concentrations, and tested the sensitivity and specificity of ddPCR. The K. pneumoniae primers and probe exhibited no cross-reactivity with 13 other pathogens, indicating that the ddPCR was highly specific to K. pneumoniae. The ddPCR had a sensitivity of 1.1 copies/μL, compared with 10 copies/μL for real-time PCR. This is in accord with the findings of previous studies that ddPCR is more sensitive than real-time PCR (31, 32). Yang et al. developed the ddPCR method to detect K. pneumoniae in blood samples. The limit of detection was 0.27 copies/μL, while the limit of blank was 0.16 copies/μL, which were lower than our method; both their and our ddPCR method show good analytical specificity and sensitivity (33).

The intestinal tract is a common colonization location of K. pneumoniae (34), so the load of K. pneumoniae in feces reflects the situation in the gut to some degree. For the first time, we quantitatively detected the bacterial load of K. pneumoniae in fecal samples. To evaluate the application of the ddPCR detection to fecal samples from the clinic, 103 fecal samples were collected and detected by using microbial culture, real-time PCR, and ddPCR methods. ddPCR showed a higher positive detection rate than fecal culture and real-time PCR. Furthermore, correlation analysis showed that there was a high correlation between the K. pneumoniae detection results from real-time PCR and ddPCR. The presence of residual matrix in fecal extract can significantly reduce the amplification effectiveness in PCR, which might result in an underestimating of the pathogen titer in clinical samples or a false-negative result. In our research, ddPCR was found less affected by residual matrix in fecal samples.

There are some limitations in this study. First, relatively small and homogeneous sample sizes of fecal samples were used in evaluating the clinical performance of ddPCR assay. Larger sample sizes and diverse sample types, considering factors such as geographical regions and patient populations, need to be selected in further research. Second, it is not possible to determine whether a single microbial infection or multiple microbial infections occur in a patient based solely on ddPCR detection results, as ddPCR can only detect a small number of target pathogens. Third, the clinical specificity of ddPCR is relatively low (Table S3), so when using ddPCR for detection, the correlation between the copy number of K. pneumoniae and diseases needs to be further studied, clinical symptoms or other detection result may also need to be considered for disease judgment.

Overall, copy numbers of K. pneumoniae in fecal samples can be accurately measured by ddPCR with excellent analytical specificity, sensitivity, repeatability, and reproducibility; additionally, ddPCR showed the high clinical sensitivity in clinical performance analyses and little inhibition effect by residual matrix. Thus, ddPCR has the potential to be applied for the diagnosis of K. pneumoniae in clinical fecal samples. Here, we developed a sensitive and effective method for the detection of K. pneumoniae in fecal samples by using ddPCR. It is a valuable method to identify causal pathogens and guide treatment decisions.

## MATERIALS AND METHODS

### Sample collection and culture.

The positive-control strain K. pneumoniae ATCC BAA-2146 was obtained from the China General Microbiological Culture Collection Center. Thirteen laboratory stored strains (Table 3) were included to evaluate the specificity of the K. pneumoniae ddPCR. One hundred and three stool samples were collected from the Capital Institute of Pediatrics for fecal culture and molecular diagnosis. The basic information of the children who contributed stool samples is summarized in Table S2. A 200-mg sample was taken from each fecal sample and diluted to the appropriate concentration using brain heart infusion broth, then fecal dilutions were plated on MacConkey agar plates and incubated for 16 h at 37°C. Selected single colonies were identified by matrix-assisted laser desorption-ionization time-of-flight mass spectrometry (Vitek MS system, bioMérieux, Germany).

**TABLE 3:** Bacterial strains used in the study

Strain	Source
Escherichia coli	Clinical isolate
Enterococcus faecalis	Clinical isolate
Enterococcus faecium	Clinical isolate
Serratia marcescens	Clinical isolate
Salmonella Typhimurium	Clinical isolate
Morganella morganii	Clinical isolate
Enterobacter cloacae	Clinical isolate
Burkholderia cepacia	Clinical isolate
Proteus mirabilis	Clinical isolate
Staphylococcus aureus	Clinical isolate
Pseudomonas aeruginosa	Clinical isolate
Citrobacter freundii	Clinical isolate
Acinetobacter lwoffii	Clinical isolate

### Recombinant plasmid construction.

The sequence of the *khe* gene from K. pneumoniae (accession no. CP003200.1) was downloaded from the NCBI GenBank database and synthesized by Sangon Biotech Co., Ltd. (Shanghai, China). Then, the synthetic *khe* gene was cloned into vector pUC57 to obtain recombinant plasmid pUC57-*khe*. The copy number of the plasmid was calculated using the formula: DNA copy number (copies/μL) = (6.02 × 10^23^ × plasmid concentration [ng/μL] × 10^−9^)/(fragment length [nt] × 660). Nano Drop (Thermo, MA, USA) was employed to measure the amount of DNA used in the experiment.

### DNA extraction.

All the bacterial strains were cultured in brain heart infusion broth at 37°C following a standard protocol, and then the total DNA of 2-mL bacterial cultures were extracted using a TIANamp bacteria DNA kit (TIANGEN Biotech Co., Ltd., Beijing, China). The total DNA of 200-mg fecal samples were extracted by using a QIAamp DNA stool minikit (Qiagen, Hilden, Germany) following the manufacturer’s instructions. The DNA was eluted with DNase-free water and stored at −80°C until use.

### Primers and probe.

**FIG 5:**
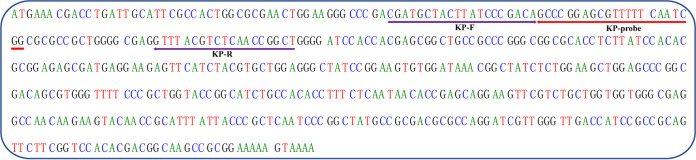
Locations of the primers and probe in the *khe* gene sequence.

The primers and probe targeting the hemolysin gene of K. pneumoniae were designed using Primer Express software (Thermo Fisher Scientific); the guanine-cytosine content and secondary structure were then assessed using OligoEvaluator software (http://www.oligoevaluator.com). The probe was labeled with 5′ FAM reporter dye (carboxyfluoroscein), and the 3′-end with a nonfluorescent quencher. Then, the designed sequences were synthesized commercially (Sangon Biotech Co., Ltd.). The sequences of the primers and probe are listed in Table 4. The locations of the primers and probe in the *khe* gene are shown in Fig. 5.

**TABLE 4:** Primers and probe for specific amplification of K. pneumoniae^*a*^

Name	Sequence (5′–3′)
KP-F	CGATGCTACTTATCCCGACA
KP-R	AGCCGGTTGAGACGTAAAC
KP-probe	6FAM-CCGATTGAAAAACGCTCCGGGC-BHQ1

a KP, klebsiella pneumoniae; F, forward primer; R, reverse primer.

### ddPCR assay.

The ddPCR assay was performed using a TARGETING ONE droplet digital PCR system (TARGETING ONE Biotech Co., Ltd., Beijing, China), including a Drop Maker and a Chip Reader. The ddPCR reaction was prepared with 2 μL of extracted DNA template, 15 μL of reaction buffer, 7.45 μL of DNase-free water, 2.4 μL of forward primer KP-F (10 μM), 2.4 μL of reverse primer KP-R (10 μM), and 0.75 μL of the probe “KP-probe” (10 μM) to give a final volume of 30 μL. The mixtures were added to a ready-to-use disposable plastic chip. Droplet generation oil (180 μL) was also added to the chip, and then the chip was loaded into a Drop Maker (TARGETING ONE) for droplet generation. Microdroplets were added into an eight-well PCR strip and PCR amplification was performed on a Veriti 96-well thermal cycler (Applied Biosystems, CA, USA). The PCR cycling parameters were as follows: denaturation at 95°C for 10 min, and 40 cycles of denaturation at 94°C for 30 s and annealing at 60°C for 1 min. Finally, the fluorescence signals were detected using a Chip Reader (TARGETING ONE) and analyzed using Chip Reader R1 software. Three replications were performed for linear regression.

To identify the ideal elongation temperature and primer/probe concentrations for ddPCR assay, the thermal gradients of 54, 56, 58, and 60°C were performed. The primer/probe concentrations of 100 nM/50 nM, 200 nM/100 nM, 400 nM/175 nM, and 800 nM/250 nM were optimized with the elongation temperature of 60°C.

### Real-time PCR assay.

The real-time PCR mixture contained the following components: 15 μL PCR master mix reagents (TIANGEN Biotech Co., Ltd.), 0.9 μL forward primer KP-F (10 μM), 0.9 μL reverse primer KP-R (10 μM), 0.6 μL KP-probe (10 μM), 2 μL DNA template and 10.6 μL of DNase-free water. All real-time PCR assays were performed using a QuantStudio 7 Flex (Applied Biosystems) instrument with the following cycling profile: initial PCR activation at 95°C for 10 min; followed by 40 cycles of denaturation at 94°C for 10 s, and annealing/extension at 60°C for 45 s. A cycle threshold (Ct) value <38 was determined to represent a positive sample.

### Estimation of the limit of detection, limit of blank, and specificity.

To analyze the limit of detection for ddPCR and real-time PCR assays, recombinant plasmid pUC57-*khe* was diluted 10-fold from 1.2 × 10^6^ copies/μL to 1.2 × 10^−1^ copies/μL with DNase-free water. To assay the limit of blank, blank samples were tested 20 times by ddPCR and real-time PCR assay. The limit of detection and the limit of blank were calculated according to the previous studies (35). For the specificity evaluation, 13 pathogens’ DNA (Table 1) were tested, the DNA of K. pneumoniae strain ATCC BAA-2146 was used as the positive control, and DNase-free water as the negative control.

### Repeatability and reproducibility of ddPCR assay.

For repeatability evaluation, 10-fold dilutions of recombinant plasmid pUC57-*khe* were examined in triplicate in one experiment run, and the intraassay coefficients of variation (CV) was calculated. For estimating of reproducibility of ddPCR, three independent experiments were performed with these recombinant plasmid dilutions, then the interassay CV was calculated.

### Detection of ddPCR and real-time PCR inhibition by residual matrix.

To identify the inhibition effect of residual matrix, the capacity of the ddPCR and real-time PCR assays to quantify a constant amount of plasmid DNA in the presence of different quantities (2 μL to 6 μL) of fecal DNAs were compared. The same amount of DNA from K. pneumoniae strain ATCC BAA-2146 was used to spike the assays; the effects of fecal DNA were evaluated in relation to the detected signals in sample without any additional inhibitors (no-inhibition control, which consisted of DNase-free water plus K. pneumoniae ATCC BAA-2146 DNA).

### Statistical analysis.

PASS software was used for sample size calculation. IBM SPSS Statistics version 21.0 was used for statistical analyses. The correlation of different groups was assessed by Pearson correlation analysis. A *t*-test was used to compare the inhibitory effect between different groups. A *P* value less than 0.05 was considered statistically significant.

### Ethics statement.

This work was approved by the research board of the Ethics Committee of the Capital Institute of Pediatrics and was performed in compliance with the Helsinki Declaration (Ethical Principles for Medical Research Involving Human Subjects). All fecal samples used in this study are part of routine patient management without any additional collection, and all patient data were anonymously reported.
