# Supplementary material for: Detection and Quantification of Klebsiella pneumoniae in Fecal Samples Using Digital Droplet PCR in Comparison with Real-Time PCR
Source: Microbiol Spectr. 2023 Jun 12;11(4):e04249-22. doi: 10.1128/spectrum.04249-22 (PMC10433836; doi:10.1128/spectrum.04249-22)
Supplement: Supplemental file 1 — Supplemental material. Download spectrum.04249-22-s0001.pdf, PDF file, 0.1 MB [file spectrum.04249-22-s0001.pdf]

Table S1 Specificity analysis of real-time PCR assay

Strain	Ct value
K. pneumoniae ATCC BAA-2146	29.2
Escherichia coli	non
Enterococcus faecalis	non
Enterococcus faecium	non
Serratia marcescens	non
Salmonella typhimurium	non
Morganella morganii	non
Enterobacter cloacae	non
Burkholderia cepacia	non
Proteus mirabilis	non
Staphylococcus aureus	non
Pseudomonas aeruginosa	non
Citrobacter freundii	non
Acinetobacter lwoffii	non

non: not detected.

Table S2 Basic information of all children who provide fecal sample

**Baseline variable**	**Number of children (%)**
SEX	
Male	65 (63.1)
Female	38 (36.9)
AGE	
<6	6 (5.8)
6-11	29 (28.2)
12-18	68 (66.0)

Table S3 Clinical sensitivity and clinical specificity of real-time PCR and ddPCR

	clinical sensitivity (%)	clinical specificity (%)
ddPCR	100	20
Real-time PCR	98.8	55

Abbreviation: ddPCR, droplet digital polymerase chain reaction
